# The costs of dominance: testosterone, cortisol and intestinal parasites in wild male chimpanzees

**DOI:** 10.1186/1751-0759-4-21

**Published:** 2010-12-09

**Authors:** Michael P Muehlenbein, David P Watts

**Affiliations:** 1 Department of Anthropology, Indiana University, Student Building 130, 701 E. Kirkwood Ave., Bloomington, IN 47405 USA; 2 Department of Anthropology, Yale University, P.O. Box 208277, New Haven, CT 06520 USA

## Abstract

**Background:**

Male members of primate species that form multi-male groups typically invest considerable effort into attaining and maintaining high dominance rank. Aggressive behaviors are frequently employed to acquire and maintain dominance status, and testosterone has been considered the quintessential physiological moderator of such behaviors. Testosterone can alter both neurological and musculoskeletal functions that may potentiate pre-existing patterns of aggression. However, elevated testosterone levels impose several costs, including increased metabolic rates and immunosuppression. Cortisol also limits immune and reproductive functions.

**Methods:**

To improve understanding of the relationships between dominance rank, hormones and infection status in nonhuman primates, we collected and analyzed 67 fecal samples from 22 wild adult male chimpanzees (*Pan troglodytes schweinfurthii*) at Ngogo, Kibale National Park, Uganda. Samples were analyzed for cortisol and testosterone levels as well as intestinal parasite prevalence and richness. 1,700 hours of observation data were used to determine dominance rank of each animal. We hypothesized that dominance rank would be directly associated with fecal testosterone and cortisol levels and intestinal parasite burden.

**Results:**

Fecal testosterone (but not cortisol) levels were directly associated with dominance rank, and both testosterone and cortisol were directly associated with intestinal parasite richness (number of unique species recovered). Dominance rank was directly associated with helminth (but not protozoan) parasite richness, so that *high *ranking animals had *higher *testosterone levels and *greater *helminth burden.

**Conclusions:**

One preliminary interpretation is that the antagonist pleiotropic effects of androgens and glucocorticoids place a cost on attaining and maintaining high dominance rank in this species. Because of the costs associated with elevated steroid levels, dominance status *may *be an honest signal of survivorship against helminth parasites.

## Background

Lifetime reproductive success for males is usually constrained by access to fecund females (i.e., fertilizations). Male-male contest competition for mating opportunities is common in mammals, and in those species that typically form multi-male groups, one outcome of this competition is the formation of dominance hierarchies. Male dominance hierarchies occur in many primate species, and males typically invest considerable effort into attaining and maintaining high dominance rank. Monopolization of fecund females by high ranking males and/or exclusion of rivals via aggression, alliance formation or other means at times when females are most likely to conceive would be advantageous if it leads to increased reproductive success. Genetic analyses now support the argument that high dominance rank can yield reproductive payoffs in several nonhuman primate species [1-6].

Chimpanzees (*Pan troglodytes*) live in multi-male, multi-female communities. Males are philopatric; social bonds between them are strong, and they cooperate with each other in various ways within communities and also cooperate in aggression between communities. However, males also compete for mating opportunities and form dominance hierarchies, and most males invest considerable effort into striving for high rank [7]. Because chimpanzees have a fission-fusion social system and individuals can go for long periods without encountering each other, males face an additional need to assert themselves frequently towards subordinates when they are together to maintain dominance over them. Genetic data from several chimpanzee study sites indicate that alpha males and others who attain high rank generally achieve disproportionately high reproductive success, although reproductive skew is only moderate in communities with more than a few adult males [8,9].

Male dominance status is not a simple function of aggressiveness, but acquisition and maintenance of high dominance rank often involves frequent aggression, and testosterone has been considered the quintessential physiological moderator of such behavior. Testosterone can alter both neurological and musculoskeletal functions that may potentiate pre-existing patterns of aggression. As an anabolic steroid, testosterone increases basal metabolic rates and stimulates muscle anabolism, adipose catabolism and redistribution [10-12]. Testosterone increases metabolic rates in muscle cells in vitro [13], which would be useful during competitive interactions. Testosterone reduces the refractory period between action potentials throughout the stria terminalis (connecting the hypothalamus with the amygdala), which can potentiate an aggressive response [14]. Testosterone also influences the organization of typical masculinized morphological and behavioral characteristics beginning in utero [15,16].

Direct associations between testosterone, rates of aggression, and dominance rank have been identified in several species, including nonhuman primates [17,18]. Conversely, several studies have failed to demonstrate significant correlations between aggression, dominance rank and testosterone levels [19,20]. In fact, there is surprisingly little evidence that short-term changes in testosterone levels correlate with increased levels of aggression, and fluctuations in testosterone levels in healthy, eugonadal individuals over time do not necessarily predict changes in levels of aggression within individuals, human or nonhuman [reviewed in [21] and [22]]. Rather, testosterone *may *have a permissive effect, potentiating pre-existing patterns of aggression [23]. Testosterone is also more frequently associated with aggression and dominance rank during situations of social instability, such as during challenges by conspecific males for territory or access to mates, the establishment of territorial boundaries, the formation of dominance relationships, or in the presence of receptive females [24].

Testosterone *may *facilitate attainment of high rank, and thus increase reproductive success, by modifying behaviors (e.g., aggression, mate seeking, courtship, mate guarding) and physical attributes (i.e., secondary sexual characteristics and muscle anabolism). However, there are a number of costs imposed by elevated testosterone levels. These include increased metabolic rates [25,26], increased risk of prostate cancer [27], production of oxygen radicals [28], and immunosuppression [reviewed in [29]], all of which could compromise survivorship.

Testosterone, along with many other hormones, functions as a biochemical link between various somatic and reproductive traits. Trade-offs between competing functions and traits (i.e., maintenance, reproduction and growth) are fundamental to life history evolution, particularly in organisms that are constrained by limited energy supplies [30]. Because of its multiple effects, testosterone is an important endocrinological mediator of various trade-offs, particularly that between reproduction and survivorship [31,32]. More specifically, it may balance the competing demands of increased reproductive success afforded by testosterone-mediated physique, aggressive behavior, and dominance status with increased susceptibility to illness.

As Folstad and Karter [33] originally suggested, testosterone can stimulate the development and maintenance of secondary sexual characteristics while also reducing immunocompetence. Wedekind and Folstad [34] added that the suppression of the immune system by testosterone could allow for energy to be reallocated to the production of secondary sexual characteristics, particularly muscle mass in mammals [29]. The presence of elaborate secondary sexual characteristics, or other characteristics that honestly reflect health, may therefore advertise good survivability to potential mates [35]. Many morphological and behavioral characteristics appear to be honest sexual signals of immunocompetence in avian and other species. Just a few examples include tail length in peacocks (*Pavo cristatus*) [36] and barn swallows (*Hirundo rustica*) [37], badge size in house sparrows (*Passer domesticus*) [38], antler size in white-tailed deer (*Odocoileus virginianus*) [39], song length and complexity in several avian species [40], coloration in satin bowerbirds (*Ptilorhynchus violaceus*) [41], and antler symmetry in caribou (*Rangifer tarandus*) [42]. Phenotypic traits, like coloration, in male nonhuman primates may also indicate health status [reviewed in [43]], although this has yet to be adequately investigated.

Dominance rank in nonhuman primate males may be an honest indicator of immunocompetence. If testosterone is immunosuppressive, and high dominance rank is associated with high testosterone levels, then high rank may also be associated with higher parasite burden. 'Higher quality' males may be able to withstand the immunosuppressive effects of high testosterone levels, allowing them to invest in secondary sexual characteristics or behaviors dependent on androgens. Those males with greater innate disease resistance may be better able to maintain higher testosterone levels, high ejaculate quality and other traits associated with successful reproduction [44]. Lower quality males may not be able to tolerate the immunosuppressive effects or increased energetic costs of high testosterone levels [45-47]. The antagonist pleiotropic effects of androgens may thus both limit trait exaggeration and have important influences on social behavior.

Glucocorticoids are also likely important in mediating the relationships between agonistic interactions, dominance rank, reproductive function, and immunocompetence. Glucocorticoids like cortisol and corticosterone are steroids released from the adrenal cortex in response to disruption of physiological and psychological homeostasis. While this increases circulating glucose levels to facilitate physical and mental activities and basic stress responses, prolonged elevation of glucocorticoid levels can have pathological effects on cognition, growth, reproduction, immunity and other functions [48]. For example, cortisol can inhibit inflammation and allergic reactions, lymphocyte proliferation, antibody and cytokine secretion, and macrophage activity [49-51]. Glucocorticoids can inhibit gonadotropin releasing hormone release from the hypothalamus, downregulate testicular luteinizing hormone receptors, and decrease testicular steroidogenesis [52-54].

Because cortisol is released in response to various stressors, a typical assumption has been that acute and sustained social stressors associated with low dominance status would result in chronic elevations in cortisol levels in low ranking animals. In some species, cortisol levels are higher in low than high ranking individuals, whereas in other species the opposite is true [55]. The relationships between cortisol and dominance rank may depend on access to social support systems [56]. Furthermore, during times of social instability, high ranking animals will likely exhibit the highest cortisol levels, probably due to the need for increased arousal and vigilance [57].

To improve understanding of the relationships among dominance rank, testosterone and cortisol levels, and infection status in nonhuman primates, we collected fecal samples and behavioral data from an unusually large community of wild chimpanzees. Our previous work on this community indicated a significant positive association between testosterone levels and dominance rank for adult males (n = 22 animals with 67 total fecal samples; mixed model analysis controlling for age, p = 0.032) [58]. Other analyses indicated that fecal testosterone (p = 0.033) and cortisol (p = 0.020) were positively associated with parasite richness (the number of unique intestinal parasite species recovered from hosts' fecal samples) in both adult and adolescent males (n = 35 animals with 100 total fecal samples; mixed model analysis controlling for age) [59]. In the present study, we add to this research agenda by better describing the complex relationships between dominance rank, fecal intestinal parasite infections, and cortisol and testosterone levels in the adult male chimpanzees from the Ngogo population.

## Methods

### Study site and subjects

Ngogo is in Kibale National Park in western Uganda. The park is located between 0°41'N, 30°19'E and 0°13'N, 30°32'E, with a total area of approximately 750 km^2^. The Ngogo study area is about 25 km^2 ^and contains old growth, regenerating, and swamp forest, *Acanthus *scrub, and other vegetation types [60]. The field site is devoid of domestic herbivores and pets. Human observation of the chimpanzees is restricted to researchers and Ugandan field assistants, and latrines and garbage pits are used for disposal of human waste and refuse at the research camp. The chimpanzees do not enter camp, nor do they enter fields outside of the park boundaries, limiting potential contact of chimpanzees with human or domestic animal feces.

**Figure 1:**
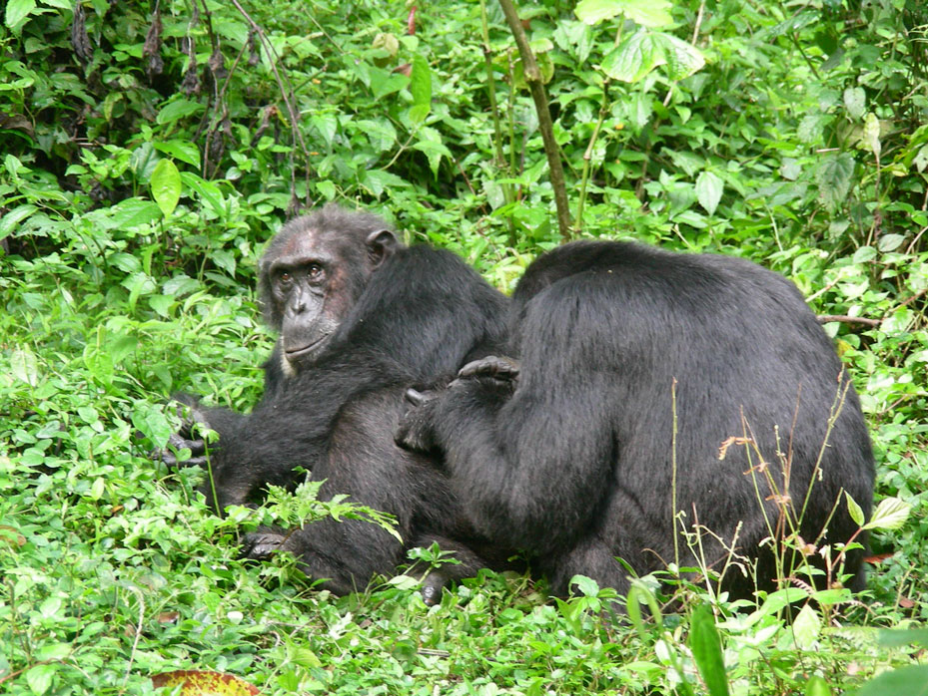
**Low ranking adult male chimpanzee grooming a high ranking male at Ngogo**.

The Ngogo chimpanzee community (Figure 1) was originally studied by Ghiglieri in the late 1970's and early 1980's [61]. Research and habituation efforts resumed at Ngogo in 1991, and have been continuous since 1995. All adult and adolescent male chimpanzees are well habituated and are observable within 5-10 m on the ground. At the time of this study, the Ngogo community had 24 adult males, 14 adolescent males and a total of approximately 150 members.

Exact ages of Ngogo community members are unknown. Adult animals were assigned to the following age categories, based on physical characteristics (noticeable teeth wear, thinning of hair, loss of muscle mass) and on the history of observations: 1 = old, 2 = prime old, 3 = prime, 4 = young prime, 5 = young. Adolescent males were classified on a different scale: 1 = closest to young adulthood (oldest adolescents), 6 = closed to juvenile stage (youngest adolescents). Age is controlled for in all statistically analyses because hormones and dominance rank usually covary with age. However, age was unassociated with intestinal parasite richness in these animals [62].

1,700 hours of observational data were collected between June and December, 2002. Most data on social behavior came from focal samples of males, but data on agonistic behavior and on the formation of coalitions by two or more males were also collected on an *ad lib *basis. Data on decided agonistic interactions ("pant-grunt" vocalizations, submissive responses to aggression, etc.) were analyzed for the presence of a linear dominance hierarchy among the 22 adult males by calculating Landau's linearity index, corrected for ties (h') in MatMan (Noldus Information Technology, Leesburg, VA, USA). For the purposes of categorical data analyses, adult males were categorized into high, medium, and low rank groups. Adult males with dominance ranks between 1 (highest) and 7 were assigned to the high group. Those ranking between 8 and 15 were assigned to the medium group, and those ranking from 6 to 22 were assigned to the low group.

### Sample collection

67 fecal samples were collected opportunistically from 22 adult male chimpanzees at Ngogo, between July and September 2002. A mean of 3.32 samples was collected per individual (range = 1 to 5). Samples from the same individual were collected on non-consecutive days (spacing between the consecutive samples ranged between 2 and 21 days). Samples were collected immediately following defecation, thus insuring positive matching of the individual with the sample. Portions of samples that might have been contaminated by soil or pooled water were not collected, nor was diarrhea. Blood and mucous were not observed in any fecal mass collected, nor did color or consistency differ significantly between masses.

Most samples were collected before 11 AM. Diurnal effects on parasite output in chimpanzees are unknown. Diurnal variation in steroid hormone secretion can be a significant concern, particularly in smaller-bodied primates such as common marmosets (*Callithrix jacchus*) [63] and tufted Capuchins (*Cebus apella nigritus*) [20]. However, diurnal variation in fecal hormone levels is probably of less concern in larger-bodied animals, such as chimpanzees (*Pan troglodytes*), due to longer gut retention time [64,65]. Fecal steroids represent long-term baseline levels with little susceptibility to minor rapid fluctuations in the hypothalamic-pituitary-gonadal axis.

A portion of each sample was preserved using Para-Pak plastic transport vials (Meridian Diagnostics, Cincinnati, OH, USA) pre-aliquoted with 10% neutral buffered formalin. A separate portion of each sample was dehydrated on an aluminum dish for approximately 2 hours at 100°C in a portable Coleman oven placed atop a kerosene stove. Following desiccation, each sample was individually packaged with silica gel and shipped back to the USA using a CDC import permit.

### Hormone analyses

For each extraction of testosterone and cortisol, a 0.3 gm sample of feces was homogenized in 4 ml methanol:acetone (8:2, v/v) and filtered with a 0.2 μm nylon centrifuge filter (Centrex MF; Scheicher & Schuell, Keene, NH, USA). The filtrate was extracted on Sep-Pak VAC C18 columns (500 mg) (Water Corp., Milford, MA, USA). An equal volume of water was added to dilute the sample, which was then layered onto a column primed according to manufacturer's instructions. The column was washed with 5 ml water, and the steroid fraction eluted with 3 ml methanol. Extraction recovery, measured by the addition of I^125 ^labeled steroid to fecal samples prior to extraction, averaged 65% for testosterone and 72% for cortisol.

The testosterone assay used reagents from the Equate Testosterone RIA kit (Binax, South Portland, ME, USA). An aliquot of each extract was reconstituted in working buffer (0.1% gelatin phosphate buffered saline) at a 1:5 dilution. ^125^I testosterone tracer (50 μl) and 100 μl antiserum (diluted 1:2) were added to 100 μl aliquots of standards (diluted 1:10 to give concentrations of 1-100 ng/dL), samples, and controls (diluted 1:10). After vortexing and overnight incubation at room temperature, 500 μl second antibody (PEG goat anti-rabbit antibody solution diluted 1:2) was added. After 20 min incubation at room temperature, incubates were centrifuged at 1500 rpm × gm for 60 min at 4°C. The supernatant was decanted and the radioactivity in the precipitant was determined by 5 min counts in a gamma counter. Sensitivity was 6 ng/dL. Cross-reactivity was 1.7% for dihydrotestosterone and less than 0.1% for all other steroids. Accuracy was tested by the addition of steroid standards to a chimpanzee extract. The mean percentage of observed concentration to expected values in the Equate testosterone assay was 91.4 ± 5.0% (n = 6).

Internal controls were run in every assay and consisted of human serum controls (male and female) provided with the Equate RIA kit along with clinical serum standards (BioRad, Hercules, CA, USA). Intra-assay variation was assessed using the mean coefficient of variation of duplicates of male controls (n = 5) and chimpanzee fecal extracts (n = 19). The mean intra-assay coefficients of variation for duplicates of male serum control (55.0 ng/dL) was 2.7%; that for fecal extract duplicates was 4.4%. Inter-assay variation for serum controls was assessed using the coefficient of variation of male and female and BioRad controls from five separate assays. The inter-assay coefficient of variation of samples was assessed using the mean of coefficients of variation for three chimpanzee samples analyzed in two separate assays. Inter-assay coefficients of variation were 4.2% for the Equate female serum control (4.8 ng/dL), 4.6% for the Equate male serum control (55.0 ng/dL), 4.2% and 7.2% for BioRad controls 1 (4.7 ng/dL) and 2 (58.2 ng/dL), and 10.1% for the three chimpanzee samples.

The cortisol assay used reagents from the Diagnostics Products Corporation Double Antibody ^125^I cortisol kit (DPC KCOD, Los Angeles, CA, USA) for serum determinations. Working buffer was distilled water. A tracer-antiserum solution was prepared by mixing equal parts of ^125^I cortisol and cortisol antiserum, and 50 μl added to 25 μl aliquots of the standards (diluted 1:10 to give concentrations of 1-50 ng/ml), samples (concentrated 10:1), and controls (diluted 1:10). Each was vortexed and incubated at 37°C. After 45 min, 250 μl of cold precipitating solution was added, and the incubates were vortexed, incubated an additional 5 min at room temperature, and centrifuged at 3000 rpm × gram for 15 min at room temperature. Following decanting of the supernatant, the radioactivity of the precipitate was determined by 5 min counts in a gamma counter. Sensitivity of the assay was 2.2 ng/ml. Cross-reactivities were 3.9% for cortisone, 3.6% for 6β-hydroxycortisol, 1.1% for corticosterone, and less than 1% for all other steroids. Accuracy, tested by the addition of cortisol standards to a chimpanzee extract, averaged 96.8 ± 2.6% (n = 5).

Internal controls were run in every cortisol assay and consisted of clinical serum standards (Bio-Rad 1, 2, 3). Intra-assay variation was assessed using the mean coefficient of variation of duplicates of BioRad controls (n = 4) and chimpanzee fecal extracts (n = 12). Mean intra-assay coefficient of variation for duplicates of BioRad controls was 2.2%. Mean intra-assay coefficient of variation for sample duplicates was 11.6%. Inter-assay variation for serum controls was assessed using the coefficient of variation of BioRad controls from five separate assays. Mean inter-assay coefficient of variation for BioRad serum control #1 (low) was 11.5%; mean inter-assay coefficient of variation for BioRad serum control #2 (medium) was 8.7%; mean inter-assay coefficient of variation for BioRad serum control #3 (high) was 5.3%.

### Parasite analyses

The formalin-fixed samples were examined using the formalin-ethyl acetate sedimentation technique [66]. Stool samples were emulsified and filtered through two layers of wet gauze into a plastic cup. The stool was washed with saline solution, placed into a 15 ml conical-bottom centrifuge tube, and centrifuged at 500 rpm × gram for 3 minutes. The supernatant was discarded, and the sediment was re-suspended in 10 ml of 10% formalin. 3 ml of ethyl acetate was added to separate the fat in the sample, and the suspension was shaken vigorously for 30 seconds. The specimen was re-centrifuged, the fat/debris plug was removed with an applicator stick, and the supernatant discarded. The remaining pellet was re-suspended using a drop of Lugol's iodine solution, and the entire pellet was examined at 10× and 40×.

Intestinal parasite infection status is often measured as parasite richness (the number of species recovered from hosts' fecal samples) or parasite intensity (the number of eggs/cysts/larva per unit mass of feces). Parasite excretion can vary dramatically within and between individuals, and parasite egg/cyst/larvae abundance in any given fecal sample may not directly correlate with the number of parasites in the chimpanzee host at any given time. Parasite excretion may not reflect the immune status of a host, although disagreement exists on this point [67]. Parasite richness probably at least reflects the ability of the host to control infections with multiple parasites at any given time. Therefore we used data only on parasite richness because we consider this a more robust measure than intensity.

### Statistical analyses

Data were entered into an Access database that was imported into SAS/STAT software (SAS Institute Inc., Cary, NC, USA). Mixed modeling (PROC MIXED) was used to examine relationships between intestinal parasite richness, dominance rank and hormone levels. Mixed modeling allowed the use of all data points, including individuals with missing observations, and avoided the need for averaging testosterone levels and parasite measures for individuals and sampling intervals. It also allowed examination of within-subject effects of continuous variables and control of within-subject covariates (age). A time-series covariance structure that did not assume equal spacing of sample intervals was used in addition to a compound symmetry covariance structure that assumed correlations remained constant. This is a reasonable assumption given the short 3-month sampling period and stability of the dominance hierarchy (see below). A time-series covariance structure accounts for unequal time periods between sequential samples as well as differences in the number of samples collected for each animal. Sampling frequency did not vary consistently with dominance rank (i.e., higher ranking animals were not sampled more frequently). However, parasite species richness significantly increased for every sequential sample taken (up to four samples) from those adult, adolescent and juvenile males sampled within the Ngogo community [62]. The average time between consecutive samples collected was 7.74 days.

Partial Spearman correlations (controlling for age) were used in addition to the mixed models. A negative correlation coefficient indicates a positive association because the highest ranking animal was ranked number one whereas the lowest ranking animal was ranked number twenty two. Level of significance was always set at 0.05.

## Results

Data on all decided agonistic interactions produced a highly significant linear dominance hierarchy (h' = 0.97, p = 0.0001; 10,000 matrix permutations). No major rank challenges occurred between male dyads during the period of sample collection. Most aggression between males took the form of charging displays. The rate at which males displayed at others increased significantly with increasing dominance rank (Spearman rank correlation; r_s _= -0.95, df = 21, p < 0.001; by convention, the highest rank is assigned a value of one). High-ranking males also engaged in more coalitionary aggression than low ranking males (r_s _= -0.81, df = 21, p < 0.001) and received more grooming than low ranking males (r_s _= -0.63, df = 21, p < 0.002).

Among the Ngogo male chimpanzees, twelve taxa of intestinal parasites (five helminth and seven protozoan) were recovered, the four most prevalent being *Troglodytella abrassarti *(97.3% of hosts), *Oesophagostomum *sp. (81.1%), *Strongyloides *sp. (83.8%), and *Entamoeba chattoni *(70.3%). The mean numbers of unique helminth and protozoan species recovered per adult individual were 2.50 and 1.00, respectively. The intestinal parasite fauna recovered from all adult and adolescent males is described in detail elsewhere [62].

Mean testosterone level for adult animals (n = 22 animals; 67 total samples) was 8.22 ng/gm (range: 2.23-14.52; S.D.: 3.40). Mean cortisol level for adult animals was 3.45 ng/gm (range: 0.57-7.57; S.D.: 1.98). Testosterone levels were positively and significantly associated with dominance rank, after adjusting for age (F = 5.51, df = 1, p = 0.032) [see also [58]]. New analyses here indicate that cortisol and dominance were *not *significantly associated (F = 0.13, df = 1, p = 0.72).

**Figure 2:**
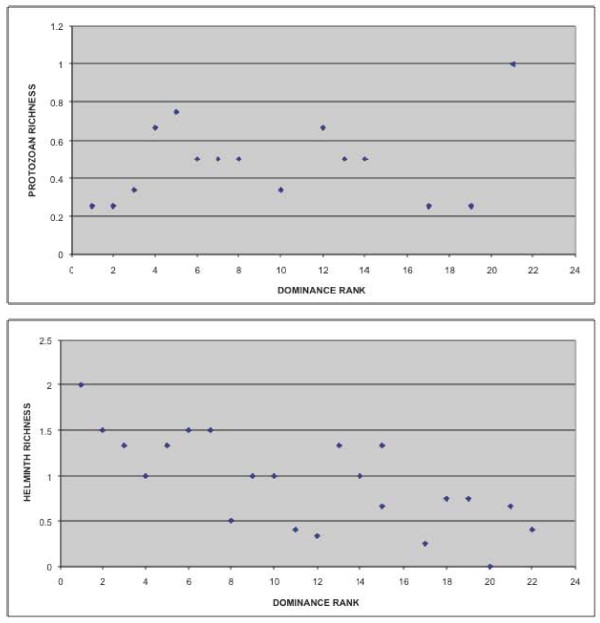
**Dominance rank by helminth and protozoan richness for each animal**. For graphic representation, parasite richness was summed across samples from each animal, and was subsequently divided by number of samples obtained from that particular animal.

We have previously shown that, when both testosterone and cortisol were placed in a mixed model controlling for age (n = 35 adult and adolescent animals; 100 fecal samples total), both testosterone (F = 4.98, df = 1, p = 0.033) and cortisol (F = 5.94, df = 1, p = 0.020) were positively associated with total intestinal parasite richness (both helminths and protozoa) [59]. New analyses here indicate that dominance rank is significantly associated with helminth parasite richness (mixed model controlling for age, F = 5.37, p = 0.034), however the association between dominance rank and protozoan parasite richness only approaches significance (mixed model controlling for age, F = 4.46, p = 0.051). For the partial Spearman correlations, dominance rank is significantly associated with total (helminth and protozoan) parasite richness (r = -0.44, p = 0.045) and helminth parasite richness (r = -0.63, p = 0.002), but *not *protozoan parasite richness (r = -0.16, p = 0.501) (Figure 2).

The use of categorized rank variables confirms these findings. Whereas the association between dominance rank category and helminth parasite richness approaches significance (mixed model controlling for age, F = 3.47, p = 0.058), there is no statistical association between dominance rank category and protozoan parasite richness (mixed model controlling for age, F = 1.11, p = 0.355). For the partial Spearman correlations, dominance rank category is significantly associated with total (helminth and protozoan) parasite richness (r = -0.47, p = 0.033) and helminth parasite richness (r = -0.60, p = 0.004), but *not *protozoan parasite richness (r = -0.24, p = 0.303)

## Discussion

Adult male chimpanzees at Ngogo exhibited a linear dominance hierarchy during the study period. Fecal testosterone levels were significantly associated (directly) with dominance rank so that higher ranking animals had higher testosterone levels. Cortisol was *not *significantly associated with dominance. Several intestinal parasite species were recovered from the fecal samples, and both testosterone and cortisol were positively associated with intestinal parasite richness (number of unique helminth and protozoan species recovered). Dominance was directly associated with helminth parasite richness. High ranking males had generally higher testosterone levels and increased helminth, but not protozoan, burden (richness) compared to lower ranking animals. To our knowledge, this provides the first analyses of the relationships among testosterone, cortisol, infection and dominance status in primates, and one of the first in wild mammals [see [68] for analysis on fecal testosterone, dominance and parasite egg counts in male Alpine ibex (*Capra ibex*)].

### Immunomodulatory actions of testosterone and cortisol

Susceptibility to infection differs among individuals, and neuroendocrine mechanisms may account for these differences. The mammalian immune responses to gastrointestinal infection are typified by a combination of phagocytosis [69,70], activation of the complement cascade and antibody responses to block cellular invasion [71], and Th-1 and Th-2 cytokine release, which facilitates gut inflammation [71-74]. Nematode infections are typically controlled via the Th-2 response with eosinophilia, goblet cell hyperplasia, mucin production, and intestinal mastocytosis [75-79]. Both testosterone and cortisol may affect these responses.

Testosterone's immunomodulatory actions appear to be primarily suppressive, increasing suppressor T cell populations, reducing T-helper cell function, inhibiting cytokine and antibody production, and impairing natural killer cell and macrophage activity [80-90]. By favoring the development of a CD4+ type-1 phenotype of peripheral lymphocytes and cytokines [91-93], elevated testosterone levels may increase susceptibility to infections that are normally cleared via the Th-2 response, like gastrointestinal infections. Not surprisingly, testosterone administration to female soft-furred rats results in reduced expulsion of the nematode *Nippostrongylus brasilnesis *[94]. Testosterone treatment in mice is also associated with increased tapeworm egg production [33] and increased susceptibility of females to *Strongyloides ratti *infection [95]. Saino and Moller [96] also identified a negative association between testosterone and parasite load in barn swallows (*Hirundo rustica*). It may be that testosterone-mediated suppression of Th-2 anti-inflammatory cytokines diminishes allergic responses that are needed to clear intestinal helminth infections. This may explain why those chimpanzees with higher testosterone level exhibit increased helminth burden.

Cortisol's immunomodulatory actions are also primarily inhibitory [[97], and see above]. In addition to chimpanzees at Ngogo, inverse associations between cortisol and immune measures have been identified in wild baboons and red colobus monkeys. In female baboons, cortisol was inversely associated with total lymphocyte levels [98]. In male baboons, cortisol was inversely associated with insulin-like growth factor I [99]. Chapman and others [100] have identified a direct association between fecal cortisol levels and nematode infection in wild red colobus monkeys (*Procolobus rufomitratus tephrosceles*) of Kibale National Park, Uganda. In these and other cases, elevated or otherwise dysregulated glucocorticoid responses to behavioral or physical stressors could result in various physical impairments, including altered lipid profiles and other cardiovascular system changes [101]. In the present study, cortisol was not significantly associated with dominance rank, but was associated with intestinal parasite richness.

### Dominance and immune functions

Relationships between dominance rank and immune measures have been identified in several species. In some, immunocompetence is lower in high status animals. For example, intestinal infections with the trematode *Genitocotyle mediterranea *were greater in dominant male European wrasse (*Symphodus ocellatus*) than in smaller, subordinate males [102]. High rank was associated with lower spleen mass and antibody levels in response to human IgG in Brandt's voles (*Lasiopodomys brandtii*) [103]. In contrast, high status individuals of other species frequently exhibit elevated immune responses relative to their subordinate counterparts. High ranking male greenfinches (*Carduelis chloris*) clear infections from Sindbis virus more quickly than lower ranking animals [104]. High ranking female dairy goats had fewer gastrointestinal parasite eggs in their feces than medium and low ranking individuals [105]. Dominant pigs exhibited higher lymphocyte proliferation to Aujeszky disease virus and the mitogens concanavalin A and phytohemagglutinin than subordinate animals [106,107].

In contrast, dominance status was unrelated to both testosterone and fecal parasite egg counts in male Alpine ibex (*Capra ibex*) [68]. Dominance status was also unrelated to anti-Seoul virus IgG responses in inoculated male Norway rats (*Rattus norvegicus*) [108].

Studies of nonhuman primates also provide mixed results. In three small, mixed sex, captive groups of chimpanzees, dominance rank was significantly negatively correlated with immunoglobulin (IgG and IgM) levels [109]. Dominant male longtailed macques (*Macaca fascicularis*) exhibited lower primary antibody responses to tetanus toxoid [110]. High ranking male yellow baboons at Amboseli, Kenya, had greater intestinal helminth infections than low ranking males [111]. In contrast, Muller-Graf and others [112] found no association between helminth infection and dominance rank in olive baboons (*Papio cynocephalus anubis*). Social subordinance is associated with increased louse prevalence, lower insulin-like growth factor I, and fewer circulating lymphocytes in olive baboons (*Papio anubis*) [99,101,113]. Low ranking female rhesus macaques (*Macaca mulatta*) had lower CD4+ and CD8+ lymphocyte counts than higher ranking females [114], and low ranking male longtailed macaques (*Macaca fasicularis*) were at greater risk of adenovirus infection than high ranking animals [115]. The present study suggests that high ranking adult male chimpanzees have increased helminth burden compared to low ranking males.

However, just as the relationships between dominance rank and circulating hormone levels within a species may depend on many factors, including access to social support, stability in the dominance hierarchy, and individual personality [116-121], so too should the relationships between dominance and immune status depend on several factors. Because high ranking males typically have more mating opportunities, they may be at increased risk of acquiring directly-transmitted infections [122]. Likelihood of exposure may vary with home range size, daily travel distance, and variation in social networks. Increased grooming opportunities can decrease the risk of arthropod-born diseases. Receiving grooming, in particular, may protect individuals against deleterious effects of chronic stress responses [121] and thereby promote immune status. High ranking males at Ngogo are attractive grooming partners [123] and the amount of grooming received was positively associated with rank during our study period. Increased social support can lead to decreased cortisol and catecholamine levels independently of rank [e.g., baboons: [118-120]], although that high ranking males at Ngogo received more grooming and more coalitionary support than low ranking males might help to explain why cortisol levels were not significantly correlated with rank. Personality factors, particularly sociability, can mediate disease outcomes [124,125]. Also, nutritional status is a major determinant of disease susceptibility and immunocompetence [126]. In so far as high rank confers greater access to nutritional resources, high ranking individuals should be able to bolster immune responses and withstand greater infection loads than subordinates.

## Conclusions

Our results are consistent with the supposition that high ranking male chimpanzees have higher testosterone levels and increased intestinal helminth burden (richness) compared to lower ranking animals, and that neuroendocrine mechanisms may account for rank-related differences in susceptibility to infection. Elevated testosterone levels in high ranking male chimpanzees at Ngogo might have contributed directly to suppressed immunity, and depressed mucosal immunity might have translated into increased susceptibility to multiple intestinal *helminth *infections. Elevated androgen levels might also have contributed to immunosuppression by promoting anabolism and thus decreasing the amount of energy and nutrients available for immunocompetence [29,34,127]. Such costs of dominance may constrain tenure length for alpha males.

The present study provides the first description of the complex relationships between dominance rank, testosterone, cortisol and infection status in nonhuman primates. Interestingly, protozoan parasite richness was not associated with dominance rank as expected, although it was associated with fecal testosterone and cortisol levels. Our admittedly small sample size of 67 (from 22 adult animals) prevent us from drawing any definitive conclusions, particularly in reference to potential differences in relationships between behavioral and endocrine variables with helminth output compared to protozoan. One interpretation may be that the helminth parasites (*Oesophagostomum*, *Strongyloides*, *Physaloptera*, *Probstmayria*, and *Hymenolepis*) are more difficult to control and impose greater immunological costs compared to the protozoan parasites (*Entamoeba coli*, *Entamoeba hartmanni*, *Entamoeba chattoni*, *Endolimaz*, *Iodamoeba, Blastocystis*, and *Troglodytella abrassarti*) recovered here. Future studies that utilize year-round sampling, particularly during and after rank reversals or other significant social challenges, would function to confirm our results.

In general, causal relationships among behavioral, endocrine and health variables remain equivocal. Some males may attain high ranks because of high testosterone levels that facilitate status-seeking behavior, but that ultimately result in higher parasite loads. Males that are more disease resistant may also be relatively good competitors and likely to achieve high status. The reverse could also hold; for example, *Taenia crassiceps *infection in male mice decreases the likelihood that a male will achieve high dominance status [128].

Alternatively, dominant animals may exhibit elevated testosterone levels, and thus higher helminth parasite burdens, as a result of gaining high status. Hormones change the likelihood of expressing a behavior in a certain social context; simultaneously, behavior affects hormone levels. This two-way relationship is evident in human males during competition: testosterone levels increase in anticipation of impending competition, then continue to rise in winners but may decline in losers [21,129], although the magnitude of winning and losing effects depends partly on whether individuals attribute the outcome to their own efforts or to external causes [22]. Similarly, hormones, status competition and immunocompetence may influence one another in chimpanzees and other species. Further work on other species, particularly nonhuman primates, is warranted. Documenting the contribution of infection status to fitness in this and other populations will prove valuable in both behavioral ecology and ecological immunology.

## Competing interests

The authors declare that they have no competing interests.

## Authors' contributions

MPM conceptualized and designed the study, collected fecal samples, conducted the parasitological analyses, performed the statistical analyses, interpreted the results and drafted the manuscript. DPW collected fecal samples, collected and analyzed the behavioral data, and assisted with interpretation of results and manuscript preparation. Both authors read and approved the final manuscript.
